# UNI‐494 treatment improves measures of renal dysfunction and cardiac pathology in male rats receiving L‐NAME and angiotensin II

**DOI:** 10.14814/phy2.70634

**Published:** 2025-10-29

**Authors:** Fiona Jing Min Ho, Clarice Jing Rou Siow, Hayat Aljaibeji, Miao Ding, Richard N. Mitchell, Satya Medicherla, Guru Reddy, Shalabh Gupta, Gordon H. Williams, Jose R. Romero

**Affiliations:** ^1^ Division of Endocrinology, Diabetes and Metabolism Brigham and Women's Hospital/Harvard Medical School Boston Massachusetts USA; ^2^ Department of Pathology Brigham and Women's Hospital, Harvard Medical School Boston Massachusetts USA; ^3^ Unicycive Therapeutics Inc. Los Altos California USA

**Keywords:** albuminuria, angiotensin II (ANG II), cardiac damage, cardiorenal disease, CVD (cardiovascular disease), kidney injury molecule (KIM‐1), nicorandil, N‐omega‐nitro‐L‐arginine methyl ester (L‐NAME)

## Abstract

Mitochondrial dysfunction is essential in the pathophysiology of both cardiovascular disease (CVD) and chronic kidney disease (CKD). Nicorandil, a nicotinamide nitrate derivative, has been used for years as a cardioprotective agent. It binds to sulfonylurea receptors, activating mitochondrial ATP‐sensitive potassium channels (MitoK_ATP_) and functioning as a Nitric Oxide (NO) donor. However, its clinical use is limited by gastrointestinal complications. UNI‐494 is a novel nicotinamide ester derivative and selective MitoK_ATP_ channel activator that reverses mitochondrial dysfunction by closing the mitochondrial permeability transition pore (mPTP) and has renoprotective effects. However, its impact on preclinical models of cardiac and renal disease is unknown. Rodents given *N* ^ω^‐nitro‐L‐arginine methyl ester (L‐NAME) to suppress NO synthase and angiotensin II (AngII) show substantial cardiac and renal damage with significant increases in blood pressure. We hypothesize that mitochondrial dysfunction contributes to the pathophysiology of cardiorenal damage in the L‐NAME/AngII rat model and that UNI‐494 would improve cardiovascular and renal parameters. We studied the in vivo impact of UNI‐494 on cardiorenal damage in the L‐NAME/AngII rat model. Treatment with UNI‐494 significantly reduced L‐NAME/AngII‐induced albuminuria, KIM‐1 levels, and cardiac injury, with no significant effect on blood pressure. Our data suggest that cardiorenal damage can be prevented by treatment with UNI‐494.

## 1 INTRODUCTION

Cardiovascular disease (CVD) is considered the leading cause of death worldwide. Effective CVD treatment is, in part, hampered by the complex interaction of multiple systems and signaling pathways. For example, CVD is commonly linked to chronic kidney disease (**CKD**) through pathways that remain unclear (Jankowski et al., 2021; Kotwal & Perkovic, 2024). Mitochondrial dysfunction leading to increased mitochondrial reactive oxygen species (ROS) production and inflammatory responses is essential in the pathophysiology of CVD and CKD (De Giorgi et al., 2002; Zong et al., 2024). Inflammation and ROS increase mitochondrial permeability transition pore (mPTP) activity (Hunter & Haworth, 1979; Kinnally et al., 2011), leading to mitochondrial dysfunction and apoptotic/necrotic cell death (Kent et al., 2021; Zorov et al., 2000), as observed in a broad range of chronic conditions, including CKD, CVD, hypertension, and inflammation‐driven fibrosis (Irazabal & Torres, 2020; Mendez‐Barbero et al., 2023; Zong et al., 2024).

Nicorandil (N‐[2‐(nitro‐oxy) ethyl]‐3‐pyridine carboxamide) is a nicotinamide ester used for years as a cardioprotective agent approved for treating angina in Europe and Japan, but not in the United States (Ahmed, 2019). It increases coronary flow and has cardioprotective effects mediated via two principal mechanisms (Barbato, 2005; Ishida et al., 2004). First, it binds to sulfonylurea receptors, selectively activating mitochondrial ATP‐sensitive potassium channels (MitoK_ATP_), thereby reducing ROS production and reversing mitochondrial dysfunction by closing the mPTP. Second, it increases systemic venous and coronary vasodilation through a nitric oxide (NO) and cGMP effect, as it contains a nitrate moiety and functions as an NO donor (Schade et al., 2010). However, gastrointestinal side effects and rapid absorption and elimination have limited its clinical use (Lee et al., 2015).

UNI‐494 is a novel nicotinamide ester derivative and a selective MitoK_ATP_ channel activator that reverses mitochondrial dysfunction by closing the mPTP. UNI‐494 dose‐dependently reduced functional markers of renal injury [serum creatinine (sCr), blood urea nitrogen (BUN), albumin to creatinine ratio (ACR)]; the proximal tubular injury marker, urinary neutrophil gelatinase‐associated lipocalin (uNGAL); and proximal tubular damage scores in a rat model of acute kidney disease/injury (AKI)—the ischemia–reperfusion injury rat model (Medicherla et al., 2024a, 2024b). However, the effects of UNI‐494 in chronic preclinical models having both cardiac and renal disease are unknown.

We and others have documented substantial cardiac and renal damage with a significant increase in blood pressure in rodents exposed to a liberal salt diet and treatment with *N* ^ω^‐nitro‐L‐arginine methyl ester (L‐NAME; CAT # AAH6366606, Fisher Scientific, Waltham, MA) to suppress NO synthase for 14 days and angiotensin II (AngII; CAT # AAJ60866LB0, Fisher Scientific, Waltham, MA), a potent vasoconstrictor at a suppressor dose, for the last 3 days before euthanasia (Huang et al., 2023; Polichnowski et al., 2011; Rocha et al., 2000; Shukri et al., 2018). L‐NAME/AngII induces mitochondrial dysfunction, leading to increased oxidative stress and ROS production (Hamilton et al., 2016). Thus, this model shows cardiac and renal injury like that observed in humans with heart failure and resistant hypertension, namely low NO and modestly increased AngII.

We hypothesized that mitochondrial dysfunction contributes to the pathophysiology of cardiorenal damage in the L‐NAME/AngII rat model and that activation of the MitoK_ATP_ would improve cardiovascular and renal parameters in this model. We, therefore, characterized the in vivo impact of UNI‐494 on cardiac and renal damage in the L‐NAME/AngII rat model.

## 2 METHODS

### 2.1 Study approval

This study protocol was approved by the Institutional Animal Care and Use Committee (IACUC) at the Brigham and Women's Hospital (BWH), and the approval number is 2016N000387.

### 2.2 Rodents

Male Wistar rats (*n* = 64) were 8–10 weeks old and weighed ~200–220 g (Charles River Lab, Wilmington, MA, USA). Rats were maintained with unlimited access to a liberal salt diet (Purina, St. Louis, MO) and water ad libitum and housed in the BWH animal facility in a 12‐h light/dark cycle at 22 ± 1°C ambient temperature. They were allowed to acclimatize for 1 week before study initiation.

### 2.3 UNI‐494 formulation

Fresh UNI‐494 dosing solutions for 20 mg/kg/po (low dose) and 40 mg/kg/po (high dose) groups were prepared daily in 1% carboxy methyl solution (1% CMC) and dosed in 5 mL/kg dosing volumes to rats by gavage. These doses were determined based on our previous studies, which used 50 mg/kg and showed significant improvements in proximal tubular damage scores in the AKI rat model (Medicherla et al., 2024b). The active ingredient of nicotinamide adenine dinucleotide in UNI‐494 for the 20 mg/kg group is 9.2 mg/kg, and the 40 mg/kg group is 18.4 mg/kg.

### 2.4 Experimental study protocol

As previously reported, the cardiac pathology and renal injury were caused by treating rats with an inhibitor of NO synthase, L‐NAME, for 14 days, plus AngII on days 11 through 14 (Huang et al., 2023; Rocha et al., 2000; Shukri et al., 2018). In this model, blockade of the mineralocorticoid receptor (MR) substantially reduces cardiac and renal injury (Huang et al., 2023; Polichnowski et al., 2011; Rocha et al., 2000; Shukri et al., 2018). Thus, we used treatment with eplerenone (AstaTech, Bristol, PA, USA, CAT # 34998), an MR antagonist, as a positive control for our study.

The experimental study for each cohort was initiated on day 0 and completed on day 15. Five days before the study initiation, all rats started receiving a liberal salt diet (1.6% Na^+^, 1.1% potassium [Purina, Scott Pharma, Marlborough, MA, USA, CAT # 26661]). They received this diet for the duration of the study. One day before study initiation, rats were randomized to the following conditions for 14 days: (1) the control group received only liberal salt; (2) L‐NAME/AngII treated group received L‐NAME (40 mg/kg/day in drinking water) and AngII (225 μg/kg/day for days 12–14 only); (3) L‐NAME/AngII/eplerenone treated group received L‐NAME (0.2 mg/mL in drinking water), AngII (225 μg/kg/day for days 12–14 only), and eplerenone (100 mg/kg/day by gavage); (4) L‐NAME/AngII/UNI‐494 Low Dose treated group received L‐NAME (0.2 mg/mL in drinking water), AngII (225 μg/kg/day for days 12–14 only), and UNI‐494 (20 mg/kg/day by gavage); (5) L‐NAME/AngII/UNI‐494 High Dose treated group received L‐NAME (0.2 mg/mL in drinking water), AngII (225 μg/kg/day for days 12–14 only), and UNI‐494 (40 mg/kg/day by gavage). UNI‐494 was provided by Unicycive Therapeutics (Los Altos, CA). AngII was administered via osmotic minipumps (Alzet minipump—model 2001, Alza Corp., Palo Alto, CA, USA), implanted subcutaneously in each rat under isoflurane anesthesia.

### 2.5 Blood pressure measurements

Systolic and diastolic blood pressure (SBP; DBP) were measured in restrained, conscious rats as previously reported by our group using the tail‐cuff plethysmography system (CODA High Throughput System, Noninvasive Blood Pressure System, CODA‐HT8; Kent Scientific Corp., Torrington, CT) (Huang et al., 2023). Briefly, rats were kept warm for 10 min and allowed to rest quietly before the same individual took and managed blood pressure measurements. During blood pressure measurements, the trained rats were placed in a restraining holder (KENT Scientific) on a heating platform to maintain body temperature at 37 ± 1°C. A total of 35 cycles were run for each animal. All results were validated using validation software (KENT Scientific), and a minimum of 10 valid cycles were analyzed. Blood pressure was measured in each rat on Day 14 after AngII administration. All blood pressures were measured between 8 and 10 am.

### 2.6 Tissue collection

All rats were euthanized under anesthesia with isoflurane. Blood samples were collected via heart puncture, and hearts were immediately excised. The upper one‐third of the heart was placed in cassettes, kept in 10% formalin overnight, transferred to 70% ethanol until paraffin embedding, sectioning, and staining by the Harvard Medical Area Core Management System/BWH for histopathological processing. The tissue was stained with hematoxylin and eosin for pathology assessment. The scoring was performed by an independent pathologist who was unaware of the study design.

### 2.7 Urine analysis

Rats were placed in metabolic cages for 24 h, from Day 13 through Day 14, with access to food and water ad libitum; 24‐h urine samples were collected daily. Urinary albumin levels were measured using an antibody‐based test and rat‐specific ELISA assay (Cat# 41‐ALBRT‐E01, Alpco Diagnostics, Salem, NH).

Kidney injury molecule‐1 (KIM‐1) levels, a biomarker of proximal tubular injury, were measured in 24‐h urine using an ELISA assay kit (Cat# RKM100, R&D Systems, Minneapolis, MN, USA) in pg/mL, according to the manufacturer's instructions.

### 2.8 Heart histopathologic evaluation

As previously described, cardiac tissue, including the ventricle, was fixed and embedded in paraffin blocks for histopathologic analysis (Huang et al., 2023; Rocha et al., 2000; Shukri et al., 2018). Briefly, the tissue was stained with hematoxylin/eosin for light microscopic analysis. Each heart section was evaluated by a pathologist who was blinded to the treatment. Myocardial injury, defined by myocyte eosinophilia and nuclear drop‐out, or frank necrosis, was expressed numerically (0–4) as a percentage of involved myocardium on a scale from 0% to >30%.

### 2.9 Statistical analyses

Data were analyzed with GraphPad Prism V. 10.4.0. Data are presented as the means ± standard deviation (SD). For normally distributed data, the difference between the means of the groups was tested by one‐way ANOVA. *p* Values less than 0.05 were considered statistically significant.

## 3 RESULTS

The primary outcomes of this study were signs of cardiac and renal damage. Microalbumin and KIM‐1 levels in 24‐h urine collections were the biomarkers to assess renal damage.

### 3.1 Urine microalbumin

**FIGURE 1:**
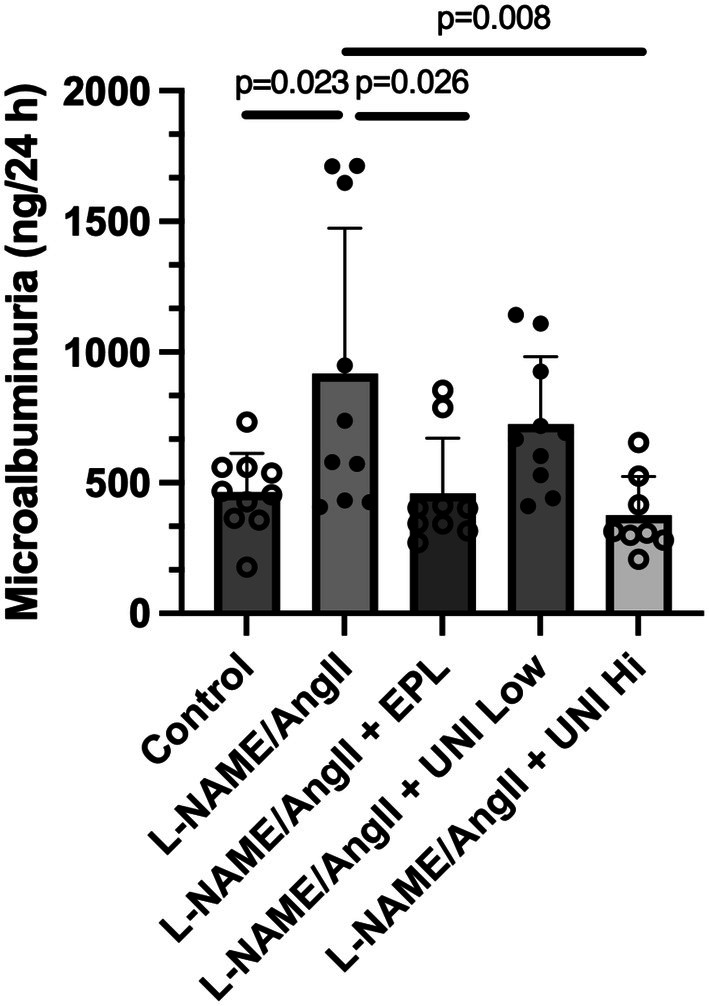
Treatment with UNI‐494 reduces the L‐NAME/AngII‐induced increase in 24‐hour urinary microalbumin. As detailed in Section 2, the Low‐Dose treated group received 20 mg/kg/day of UNI‐494 (Uni Low). The High‐Dose treated group received 40 mg/kg/day of UNI‐494 (Uni Hi). Data show the mean ± SD. *N* = 8–10 rats/group. Eplerenone (EPL); UNI‐494 (UNI).

The 24‐h urinary microalbumin was determined on Day 13, during the third day of AngII treatment (Figure 1). Compared to control rats treated only with the vehicle, those treated with L‐NAME/AngII had a doubling of microalbumin (*p* = 0.023). As anticipated, the microalbumin levels were significantly reduced in rats treated with L‐NAME/AngII and the MR antagonist eplerenone (adjusted *p* = 0.026) compared to the positive control (L‐NAME/AngII alone). The level observed was indistinguishable from the control group. Similar to the positive treatment group (eplerenone group), UNI‐494 reduced the microalbumin to the same extent in a dose–response fashion, with the rats receiving the high dose UNI‐494 having a urine microalbumin significantly lower than those rats receiving L‐NAME/AngII alone (adjusted *p* = 0.008)—a level not significantly different than the Control or Eplerenone groups.

### 3.2 Urine KIM‐1

**FIGURE 2:**
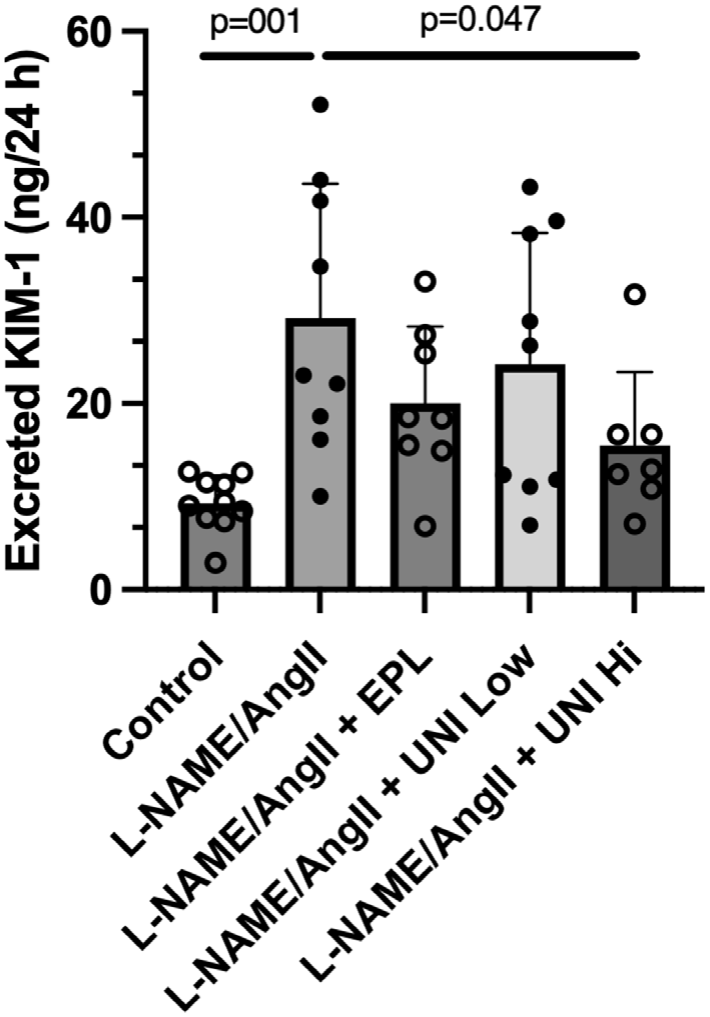
Treatment with UNI‐494 lowers L‐NAME/AngII‐stimulated 24‐hour urinary KIM‐1 levels. Data show the mean ± SD. N = 8–10 rats/group.

The 24‐hour urinary KIM‐1 was determined on day 13 during the third day of AngII treatment (Figure 2). Compared to Control rats treated only with the vehicle, those treated with L‐NAME/AngII had a tripling of KIM‐1 levels (adjusted *p* = 0.001). As anticipated, in rats treated with L‐NAME/AngII and eplerenone, KIM‐1 levels were reduced (adjusted *p* = 0.001) compared to the positive control (L‐NAME/AngII alone). Like the eplerenone treatment group, UNI‐494 reduced KIM‐1 levels in a dose–response manner, with the decline in KIM‐1 levels in the high‐dose UNI‐494 group reaching statistical significance (adjusted *p* = 0.047) (Figure 2). The KIM‐1 levels observed in the eplerenone and high‐dose UNI‐494 groups were higher but statistically indistinguishable from those in the Control group.

### 3.3 Body, kidney, and heart mass

We measured body mass in each rat at the time of sacrifice on Day 15 and summarized the data into the five treatment groups (Figure S1). No significant differences in body mass were observed between the groups.

We measured kidney mass in each rat at the time of sacrifice on Day 15 and summarized the data into the five treatment groups. Kidney mass was significantly greater in the L‐NAME/AngII than in the Control group (adjusted *p* = 0.013). While kidney mass in the three other groups was smaller than that of the L‐NAME/AngII group and greater than that of the Control group, no statistically significant differences were observed (Figure S2).

We measured the heart mass in each rat at the time of sacrifice on Day 15 and summarized the data into the five treatment groups. Heart mass was significantly greater in the L‐NAME/AngII group than in the Control group. Heart masses in the three other groups were smaller than those of the L‐NAME/AngII group and more significant than those of the control group. However, no statistically significant differences were observed (Figure S3).

### 3.4 Cardiac injury

**FIGURE 3:**
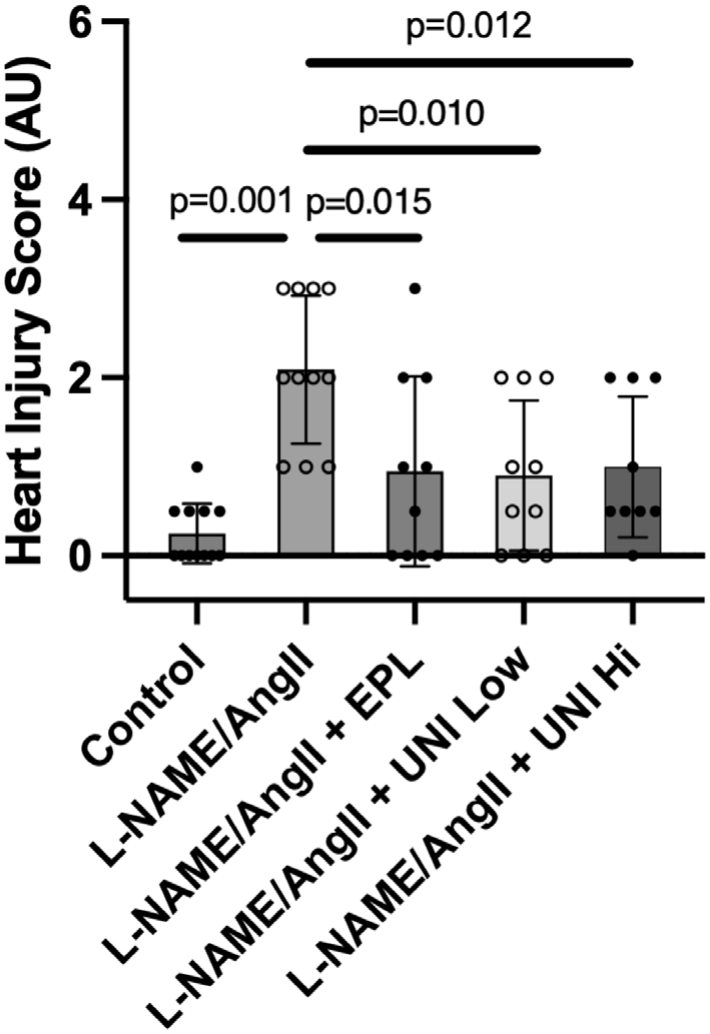
Treatment with UNI‐494 lowers L‐NAME/AngII‐induced cardiac injury. The cardiac injury score was assessed by an investigator blinded to the study design, as described in the Section 2. Data show the mean ± SD. *N* = 8–11 rats/group.

All groups receiving L‐NAME/AngII had higher inflammation scores than the Control group. The L‐NAME/AngII group increased their score significantly by over 8‐fold. All three treatment groups halved their treatment scores compared to the L‐NAME/AngII alone group (Figure 3).

### 3.5 Blood pressure

**FIGURE 4:**
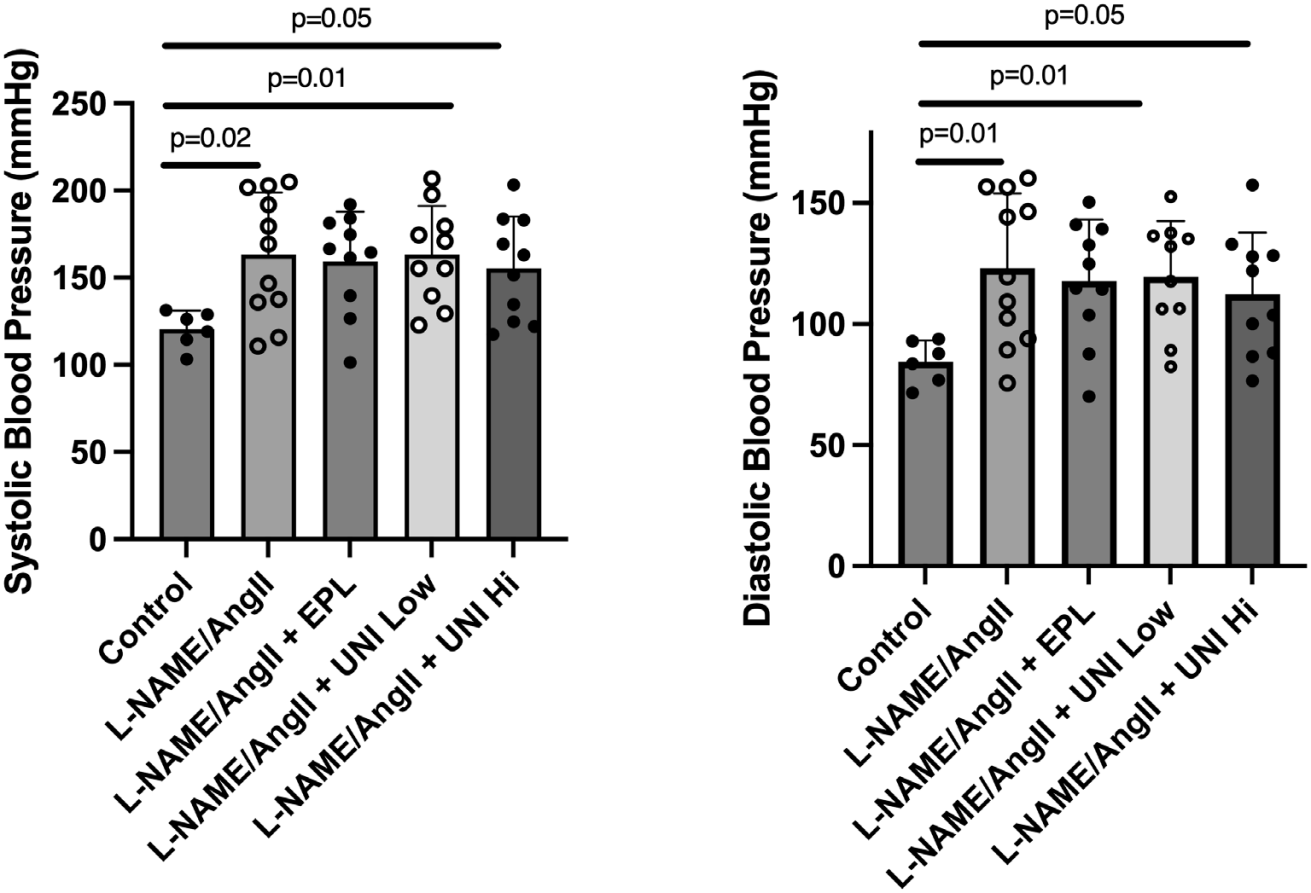
Treatment with UNI‐494 does not significantly affect the increases in systolic and diastolic blood pressure induced by L‐NAME/AngII stimulation. Blood pressure was determined by tail‐cuff as described in Section 2. Data show the mean ± SD. *N* = 8–11 rats/group.

The systolic and diastolic blood pressures were higher in all treatment groups than in the Control group (Figure 4). However, they did not differ significantly from each other.

## 4 DISCUSSION

Our results support our underlying hypothesis. Treatment with UNI‐494 significantly reduced L‐NAME/AngII‐induced renal injury (i.e., reduced KIM‐1 and albuminuria) and cardiac injury. Increases in blood pressure following administration of L‐NAME together with AngII were not modified by UNI‐494, supporting the hypothesis that in this model, the protective effects on cardiac and renal parameters were not mediated via a reduction in blood pressure. In addition, the impact of UNI‐494 was not mediated by NO synthase activation, as these cardiorenal protective effects were observed in the presence of L‐NAME. Thus, our data suggest that, like nicorandil, the nicotinamide ester derivative UNI‐494 activates MitoK_ATP_ channels, leading to a reversal of mitochondrial dysfunction and the prevention of cardiorenal damage in the L‐NAME/AngII rat model.

The current study was performed in an established model of MR‐mediated renal damage. Increased aldosterone contributes to MR activation, leading to mitochondrial dysfunction (Tsai et al., 2024). MR activation acts at several points in the onset and progression of renal dysfunction, specifically at the proximal tubule, glomerulus, and vasculature level. MR activation induces glomerular podocyte injury, thereby disrupting the glomerular filtration barrier, leading to proteinuria. Conversely, MR blockade reduces podocyte damage and proteinuria (Lin et al., 2010; Shibata et al., 2007). Our results suggest a similar role for UNI‐494, as observed with eplerenone (reduction of KIM‐1 levels, albuminuria, and cardiac injury), implying that MitoK_ATP_ activation affects both the proximal tubules and the glomerulus. These findings support and extend the growing data showing that MR antagonists may be important therapeutic agents in cardiorenal disease (Pandey et al., 2022) and that nicotinamide (e.g., nicorandil or UNI‐494) treatment improves cardiac and renal function in patients with cardiorenal syndrome (Du et al., 2021). Our results raise the possibility that UNI‐494 and eplerenone may have additive or synergistic effects when administered together in this model.

AngII is a potent vasoactive molecule and an important aldosterone secretagogue. AngII and aldosterone regulate blood pressure and stimulate the production of ROS, contributing to mitochondrial dysfunction. The L‐NAME/AngII rodent model is characterized by myocyte and vascular smooth muscle cell necrosis, accompanied by increased myocardial inflammatory processes and damage to the coronary arteries (Oestreicher et al., 2003). We documented that eplerenone blocks the MR and significantly reduces cardiovascular complications in this model, but does not reduce blood pressure (Rocha et al., 2000; Shukri et al., 2018). Notably, we now demonstrate that UNI‐494, similar to eplerenone, was effective in blocking cardiac damage while being ineffective in lowering blood pressure in this model.

KIM‐1 is primarily expressed in proximal tubular cells. Its expression is increased in proximal tubular cells following renal ischemia/reperfusion. It is considered an early biomarker of acute kidney injury and a predictor of chronic kidney injury and cardiovascular disease (Du et al., 2021; Geng et al., 2021; Huang et al., 2023). Our results suggest that proximal tubule KIM‐1 expression is reduced by treatment with UNI‐494, implying that in this model, increased permeability of the mPTP promotes proximal tubular cell damage and indicates that UNI‐494 activates MitoK_ATP_, reducing tubulointerstitial damage. These findings expand on our earlier data (reduced sCr, BUN, ACR, uNGAL, and renal damage scores) in an ischemia/reperfusion injury rat model of AKI (Medicherla et al., 2024a, 2024b).

Our study suggests that targeting mitochondrial dysfunction in heart failure, CVD, and CKD may significantly improve clinical outcomes and, as such, provides an essential rationale for developing clinical trials targeting mitochondrial dysfunction in these patients, for example, using UNI‐494. Our study controlled for environmental and other variables that could confound the interpretation of our results. Specifically, our studies were conducted under standardized conditions, including a fixed time of day for blood pressure measurements, plasma and urine collection, and a controlled sodium and potassium intake, as well as a blinded cardiac assessment. However, our study has limitations. First, we only studied male rats, so biological sex comparisons were not done. However, because we have documented increased cardiorenal damage in female rats compared to male rats in this model (Shukri et al., 2018), we hypothesize that UNI‐494 may have a more substantial protective effect in female rats than in male rats. Second, our study shows excellent prevention efficacy for UNI‐494. Consequently, studies in CVD and renal models of damage reversal are now needed. Third, while our results strongly suggest a dose‐dependent improvement in cardiorenal damage with UNI‐494 in this model, the direct effect of UNI‐494 on mitochondrial function and its specific mechanism(s) of action were not established. However, our results showing improvements in KIM‐1 levels suggest an essential role for mitochondrial function/ROS production in proximal tubular cells as an important mechanism(s) driving the beneficial effects of UNI‐494; results that are consistent with previous observations, including improved uNGAL levels following treatment with UNI‐494 in an AKI model. In addition, our studies provide an important rationale for further exploration to clarify the mechanism(s) driving the beneficial cardiorenal effects of UNI‐494 in this damage model and a damage reversal model.

## 5 CONCLUSION

The MitoK_ATP_ activator UNI‐494 reduced L‐NAME/AngII‐induced increases in KIM‐1 (a marker of proximal tubule damage), albuminuria (a marker of glomerular damage), and cardiac injury score. These cardiorenal effects of UNI‐494 were independent of blood pressure or NO effects.

## AUTHOR CONTRIBUTIONS

FJMH, CJRS, SM, GR, SG, GHW, and JRR developed the study concept. The protocol was designed, and approvals were obtained and activated by FJMH, CJRS, GHW, and JRR. MD and HA performed the animal studies, including blood pressure assessments, immunoassays, and data entry, which GHW and JRR approved. RNM performed the cardiac tissue assessment. RNM, GHW, and JRR performed data analyses and interpretations. GHW and JRR wrote the first draft of the article. All authors reviewed and approved the final draft before submission.

## FUNDING INFORMATION

This work was supported in part by a Grant‐in‐Aid to JRR and GHW from Unicycive Therapeutics, Inc., Los Altos, CA, who also supplied UNI‐494 for this study, and the Brigham and Women's Hospital—Brigham Research Institute—Research Excellence Fund, awarded to JRR.

## CONFLICT OF INTEREST STATEMENT

Authors SM, GR, and SG are employees of Unicycive Therapeutics Inc. and received financial support from Unicycive Therapeutics Inc. during the conduct of this study. GHW was a consultant for Unicycive Therapeutics Inc. All other authors declare that no conflict of interest could be perceived as prejudicing the impartiality of the research reported.

## Supporting information


Figures S1–S3.

## Data Availability

All data are available as part of the article, and no additional source data is required.
