# Supplementary material for: UNI‐494 treatment improves measures of renal dysfunction and cardiac pathology in male rats receiving L‐NAME and angiotensin II
Source: Physiol Rep. 2025 Oct 29;13(21):e70634. doi: 10.14814/phy2.70634 (PMC12571543; doi:10.14814/phy2.70634)
Supplement: Supplementary file 1 — Figures S1–S3. [file PHY2-13-e70634-s001.docx]

**SUPPLEMENTAL FIGURES**

**UNI-494 TREATMENT IMPROVES MEASURES OF RENAL DYSFUNCTION AND CARDIAC PATHOLOGY IN MALE RATS RECEIVING L-NAME AND ANGIOTENSIN II**

Fiona Jing Min Ho^1^*, Clarice Jing Rou Siow^1^*, Hayat Aljaibeji^1*^, Miao Ding^1^*, Richard N Mitchell^2^, Satya Medicherla^3^, Guru Reddy^3^, Shalabh Gupta^3^, Gordon H Williams^1^, and Jose R Romero^1^**

^1^Division of Endocrinology, Diabetes and Metabolism, Brigham and Women’s Hospital/Harvard Medical School, Boston, MA 02115

^2^Department of Pathology, Brigham and Women's Hospital, Harvard Medical School

^3^Unicycive Therapeutics Inc., 4300 El Camino Real, Los Altos, CA 94022

** Supplemental FIG 3. Heart Mass.** Data shows the mean ± SD. N=8-10/group.

** Supplemental FIG 2. Kidney Mass.** Data shows the mean ± SD. N=8-10/group.

**Supplemental FIG 1. Body Mass.** Data shows the mean ± SD. N=8-10/group.
